# Association Between Hemoglobin Glycation Index and Risk of Cardiovascular Disease and All Cause Mortality in Type 2 Diabetic Patients: A Meta-Analysis

**DOI:** 10.3389/fcvm.2021.690689

**Published:** 2021-05-28

**Authors:** Jian-di Wu, Dong-liang Liang, Yue Xie, Mei-yu Chen, Hai-hong Chen, Dan Sun, Hui-qi Hu

**Affiliations:** Department of Cardiology, The Second People's Hospital of Foshan, Foshan, China

**Keywords:** hemoglobin glycation index, glycated hemoglobin, all-cause mortality, cardiovascular disease, risk

## Abstract

**Background:** The hemoglobin glycation index (HGI) has been proposed as a marker to quantify inter-individual variation in hemoglobin glycosylation. However, whether HGI is associated with an increased risk of diabetic complications independent of glycated hemoglobin (HbA1c) remains unclear. This meta-analysis aimed to determine the association between HGI and the risk of all cause mortality and composite cardiovascular disease (CVD).

**Methods:** PubMed, and EMBASE databases were searched for related studies up to March 31, 2021. Observational studies reported associations between HGI levels and composite CVD and all cause mortality were included for meta-analysis. A random effect model was used to calculate the hazard ratios (HRs) and 95% confidence intervals (CI) for higher HGI.

**Results:** A total of five studies, comprising 22,035 patients with type two diabetes mellitus were included for analysis. The median follow-up duration was 5.0 years. After adjusted for multiple conventional cardiovascular risk factors, an increased level of HGI was associated with a higher risk of composite CVD (per 1 SD increment: HR = 1.14, 95% CI = 1.04–1.26) and all cause mortality (per 1 SD increment: HR = 1.18, 95% CI = 1.05–1.32). However, when further adjusted for HbA1c, the association between HGI and risk of composite CVD (per 1 SD increment of HGI: HR = 1.01, 95% CI = 0.93–1.10) and all cause mortality (per 1 SD increment of HGI: HR = 1.03, 95% CI = 0.96–1.10) became insignificant.

**Conclusions:** High HGI was associated with an increased risk of composite CVD and all cause mortality after adjustment for multiple conventional cardiovascular risk factors. However, the association was mainly mediating by the level of HbA1c.

## Introduction

Prevalence of type 2 diabetes mellitus (T2DM) has increased dramatically, with ~422 million adults diagnosed with T2DM worldwide (1). Cardiovascular disease (CVD) is a leading comorbidity of T2DM, contributing to the significantly increased mortality in patients with T2DM (2). Even in patients with prediabetes, the risk of all cause mortality and CVD was higher than those with normoglycemia (3, 4). Therefore, detection of biomarkers or metrics for risk stratification and guiding proper treatment in T2DM is of significant importance to relieve the global burden of T2DM (5).

Glycated hemoglobin (HbA1c) is an index that reflects the mean blood glucose (MBG) for the prior 2–3 months (6). HbA1c is considered the gold standard in evaluating of glycemic control in T2DM, broadly adopted in clinical practice and trials (7, 8). However, studies have shown considerable variation in the association between measured HbA1c and MBG. Only 60–80% of the variance in HbA1c levels could be explained by the MBG levels, while the rest is attributed to ethnic and biological factors involved in hemoglobin glycation (9, 10). To quantify such variation, the hemoglobin glycation index (HGI) was introduced by Hempe et al., which is defined as the measured HbA1c minus predicted HbA1c levels (11). The predicted HbA1c levels are calculated with equations from linear regression of HbA1c and fasting blood glucose. Therefore, individuals with high HGI values have higher measured HbA1c levels than predicted HbA1 levels from their blood glucose levels.

There has been contentious about adopting the HGI as a marker of risk stratification in patients with diabetes. It had been proposed that high HGI is associated with vascular complications in patients with and without diabetes (12–15). In a large sample cross-sectional study, high HGI was independently related to composite CVD, coronary artery disease, stroke, and peripheral artery disease after adjustment for other CVD risk factors, including HbA1c levels (16). However, the cross-sectional design limits its value to explore the causal relationship between HGI and the risk of CVD. Some prospective studies showed increased HGI was associated with an increased risk for CVD (17, 18), other studies have not found a similar association (19–21), especially after adjusting HbA1c levels. Based on these inconsistencies, we performed a meta-analysis of prospective studies to evaluate the associations between HGI and the risk of all cause mortality and CVD.

## Methods

### Search Strategy and Selection Criteria

According to the recommendations of the Meta-analysis of Observational Studies in Epidemiology group (22), we searched electronic databases, including PubMed, and EMBASE for studies up to March 31, 2021, using a combined MeSH heading and text search strategy with the following terms: “hemoglobin glycation index,” or “HGI,” and “cardiovascular disease,” “cardiovascular event,” “cardiocerebrovascular disease,” “coronary artery disease,” “coronary heart disease,” “ischemic heart disease,” “myocardial infarction,” “stroke,” “cerebral infarction,” “mortality,” or “death.” We also reviewed reference lists of included studies to identify other potential publications. The search was restricted to human studies, while without language restriction.

We included studies for meta-analysis if they met the following criteria: (1) cohort study, nest case-control study or *post-hoc* analysis of randomized controlled trials (RCTs), evaluating the associations between HGI and risk of CVD or all cause mortality; (2) the risk of endpoints were reported in high HGI compared with low HGI patients (e.g., highest tertile vs. lowest tertile), or evaluated when HGI was defined as a continuous metric; (3) multivariable-adjusted relative risks (RRs), or hazard ratios (HRs) and 95% confidence intervals (CIs) were reported; (3) the follow up duration ≥1 years. We excluded studies if: (1) they were cross-sectional studies without follow-up evaluation; (2) the association between HGI and risk of endpoints was not adjusted for other cardiovascular risk factors, and (3) identical outcomes were derived from the same population.

### Data Extraction and Quality Assessment

Two reviewers (JW and DL) screened the titles and abstracts of the retrieved reports, and reviewed the full copies of potentially relevant studies. Study information such as study design, ethnicity, participant's characteristics, sample size and proportion of female, follow-up duration, adjusted confounders, and endpoints assessment were recorded. Quality assessment of the included studies was conducted based on the Newcastle–Ottawa Quality Assessment Scale for cohort studies (23), in which an investigation was judged based on: selection (four items, one star each), comparability (one item, up to two stars), and exposure/outcome (three items, one star each), respectively. In this meta-analysis, we defined included studies as with good, fair, or poor quality if they were awarded ≥7, 4–6, or < 4 stars, respectively (24, 25).

### Data Synthesis and Analysis

The primary outcomes were the risk of composite cardiovascular events and all-cause mortality associated with HGI. The associations between the HGI and risk of outcomes were reported in different ways in the included studies, such as per standard-deviation (SD) increment in the continuous trait; or per tertiles or quartiles in HGI levels. To enable a consistent approach to the meta-analysis, we calculated the HRs per 1 SD increment in the level of HGI. If the included studies did not report effect measures as per 1 SD change, we converted the results according to the previously reported methods (26, 27).

Adjusted HRs and 95% CI were extracted for the meta-analysis. If a study reported multiple results based on different statistical adjusted models, results adjusted for the most number of variables were extracted for analysis. If RRs, but not HRs were reported, the RRs were considered as approximate HRs and included for meta-analysis (28). To explore whether the endpoints associated with HGI were mediated by the level of HbA1c, studies with both estimates of HRs before and after adjustment of HbA1c were also extracted for meta-analysis, respectively. *I*^2^ statistics were used to test heterogeneity among studies, and an *I*^2^ > 50% was considered to be with significant heterogeneity. We used the inverse variance approach with a random-effects model to combine the log HRs and corresponding standard errors (SEs). We also performed a sensitivity analysis by omitting one study and recalculating the estimates of HRs at a time. Publication bias was evaluated by inspecting funnel plots for the primary outcomes in which the natural log HR was plotted against SEs, and further tested using Egger's and Begg's tests. Subgroup analyses were performed according to participant's age, follow-up duration, and baseline CVD.

Analyses were performed using Stata 12.0 (StataCorp LP, College Station, TX, USA) and RevMan 5.3 (The Cochrane Collaboration, Copenhagen, Denmark). A *P* < 0.05 was considered as with statistical significance.

## Results

### Studies Retrieved and Characteristics

**Figure 1:**
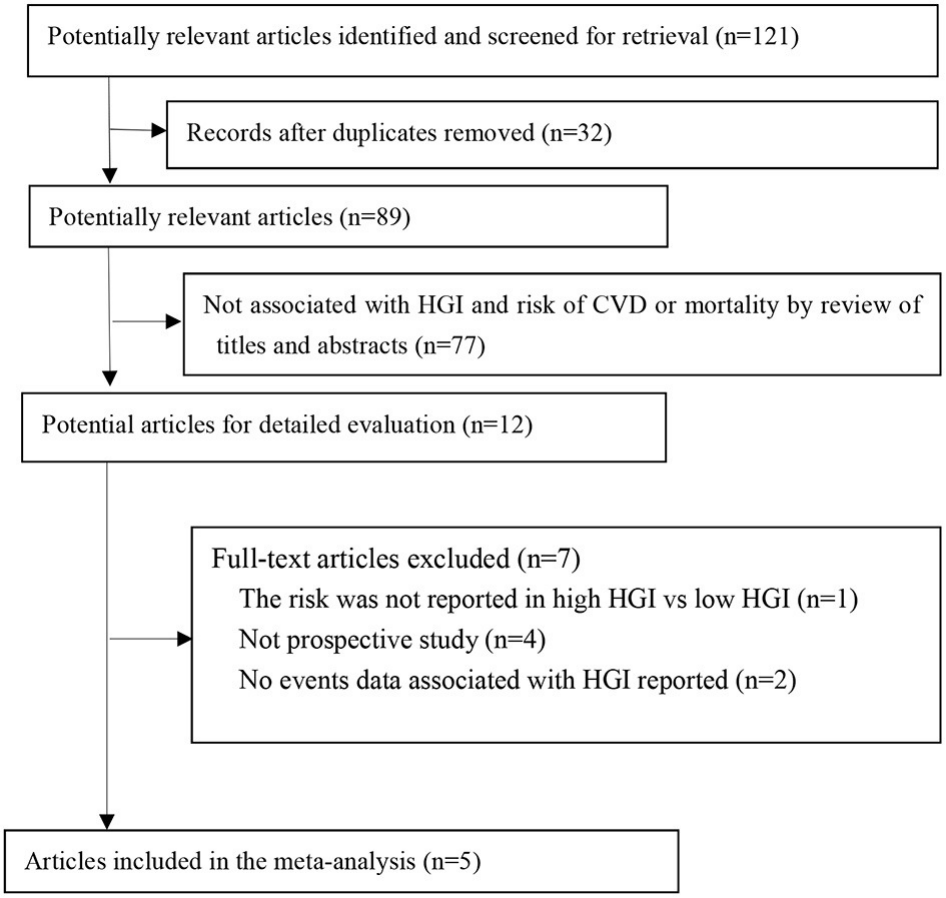
Flow of papers through review. CVD, cardiovascular disease; HGI, hemoglobin glycation index.

A total of 121 manuscripts were retrieved from the electronic databases. After screening of the titles and abstracts, 12 qualified for a full review, and five studies were included for meta-analysis finally (Figure 1) (17–21). There were 22,035 patients involved in the meta-analysis, with a median follow-up duration of 5 years.

The key characteristics of the included studies are presented in Table 1. Two studies were from Asia, one from the Netherlands, and two *post-hoc* analyses of RCTs enrolled patients from multiple countries worldwide. All the studies included patients with T2DM, while differed in with/without CVD. According to quality assessment criteria, 4 studies were graded as good quality, and only one as fair (Online Supplementary File 1).

**Table 1:** Characteristics of the included studies.

**References**	**Design**	**Country/Region**	**Patients characteristics**	**Sample (female %)**	**Age (years) (mean)**	**Follow-up duration (years)**	**Events for analysis**	**Risk factors adjusted**
van Steen et al. (21)	*Post-hoc* analysis of RCT	Multiple countries	T2DM with acute coronary syndrome	6,458 (26.9)	60.6	2.0	All-cause mortality Composite CVD Cardiovascular mortality	Age, sex, race, SBP, DBP, BMI, hemoglobin, eGFR, LDL-C, HDL-C, TG, duration of T2DM, use of antihyperglycaemic agents, history of retinopathy and smoking Additional adjustment for HbA1c level
Jin et al. (17)	Nested case-control study	China	T2DM with stable CAD	1,282 (30)	60.0	3.0	Composite CVD	Age, sex, BMI, hypertension, family history of CAD, smoke, HDL-C, non-HDL-C, Creatinine, uric acid, hs-CRP, Gensini score
Kim et al. (18)	Prospective cohort study	Korea	T2DM without CVD	1,302 (58.1)	55.5	11.1	Composite CVD CAD Stroke	Age, sex, BMI, duration of T2DM, presence of hypertension, eGFR, treatment and use of Sulfonylurea. Additional adjustment for HbA1c level
van Steen et al. (20)	*Post-hoc* analysis of RCT	Multiple countries	T2DM with a history, or a risk factor for CVD	11,083 (42.4)	65.8	5.0	All-cause mortality Composite CVD	Age, sex, race, BMI, duration of T2DM, history of macro- and microvascular events, current drinking and smoking, use of glucose-lowering drugs, use of BP-lowering drugs, SBP, DBP, hemoglobin, eGFR, LDL-C, HDL-C, TC, TG Additional adjustment for HbA1c level
Ostergaard et al. (19)	Prospective cohort study	Netherlands	T2DM	1,910 (30)	60.0	9.6	All-cause mortality Composite CVD Cardiovascular mortality Myocardial infarction Stroke	Age, sex, BMI, insulin use, duration of T2DM, non-HDL-C, eGFR, SBP Additional adjustment for HbA1c level

*BMI, body mass index; CAD, coronary artery disease; CVD, cardiovascular disease; DBP, diastolic blood pressure; eGFR, estimated glomerular filtration rate; HbA1c, Glycated hemoglobin; HDL-C, high-density lipoprotein cholesterol; LDL-C, low-density lipoprotein cholesterol; RCT, randomized controlled trial; SBP, systolic blood pressure; T2DM, type 2 diabetes mellitus; TC, total cholesterol; TG, triglycerides*.

### Association Between HGI and Prognosis in Patients With T2DM Before Adjusted for Hba1c

**Figure 2:**
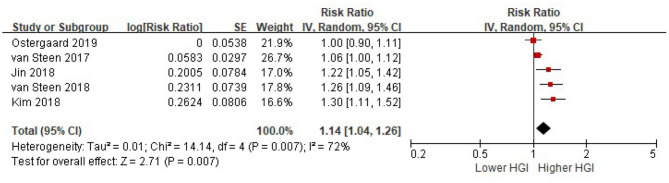
The association between HGI and the risk of composite CVD. Data were presented as the HR and 95% CI of composite CVD for per 1 SD increment of HGI, after adjusting for multiple cardiovascular risk factors (without HbA1c). CI, confidence interval; CVD, cardiovascular disease; HGI, hemoglobin glycation index.

All the included five studies provided a multi-variable adjusted risk of composite CVD associated with HGI before adjusted for HbA1c. Significant heterogeneity among all studies was observed (*I*^2^ = 72%, *P* = 0.007). Random-effects models analyses showed that per 1 SD increment of HGI was associated with a 14% higher risk of composite CVD after adjusted for multiple cardiovascular risk factors (HR = 1.14, 95% CI = 1.04–1.26, *P* = 0.007) (Figure 2). We did not find any evidence of publication bias by visual inspection of the funnel plot (Online Supplementary File 2), or using Egger's test or Begg's (all *P* > 0.05).

**Figure 3:**
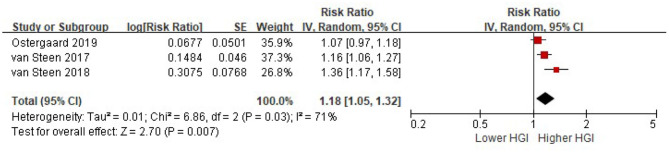
The association between HGI and the risk of all cause mortality. Data were presented as the HR and 95% CI of all cause mortality for per 1 SD increment of HGI, after adjusting for multiple cardiovascular risk factors (without HbA1c). CI, confidence interval; HGI, hemoglobin glycation index.

Three studies provided a multi-variable adjusted risk of all cause mortality associated with HGI before adjusted for HbA1c. Random-effects models analyses showed that per 1 SD increment of HGI was associated with an 18% higher risk of all cause mortality after adjusted for multiple cardiovascular risk factors, while without adjusted for HbA1c (HR = 1.18, 95% CI = 1.05–1.32, *P* = 0.007) (Figure 3).

### Association Between HGI and Risk of All-Cause Mortality and CVD After Adjusted for HbA1c

**Figure 4:**
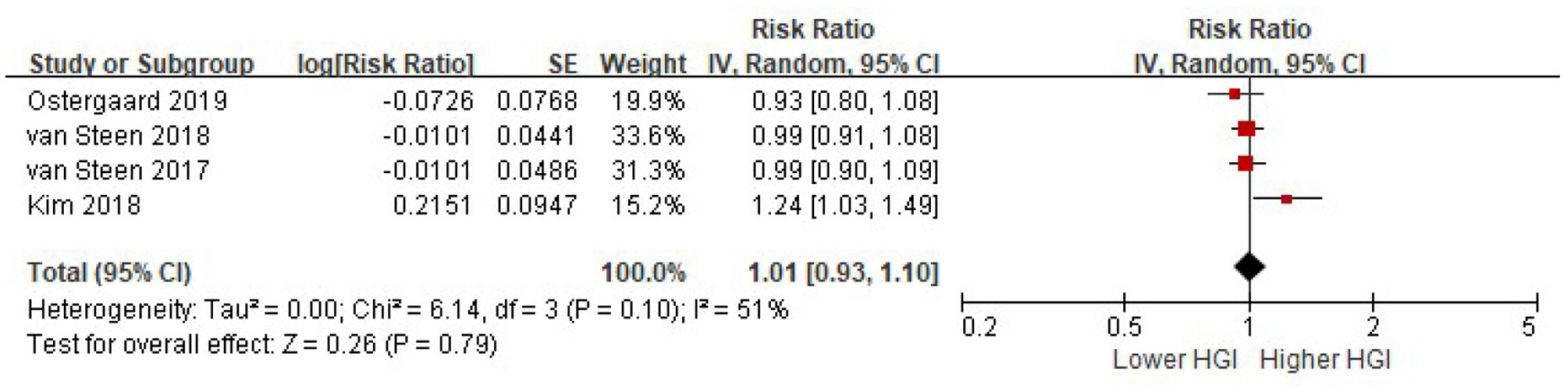
The association between HGI and the risk of composite CVD further adjusting for HbA1c. Data were presented as the HR and 95% CI of composite CVD for per 1 SD increment of HGI, after adjusting for multiple cardiovascular risk factors and plus HbA1c. CI, confidence interval; CVD, cardiovascular disease; HGI, hemoglobin glycation index.

**Figure 5:**
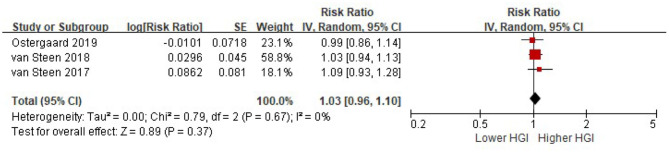
The association between HGI and the risk of all cause mortality further adjusting for HbA1c. Data were presented as the HR and 95% CI of composite CVD for per 1 SD increment of HGI, after adjusting for multiple cardiovascular risk factors and plus HbA1c. CI, confidence interval; HGI, hemoglobin glycation index.

Four studies reported the risk of composite CVD associated with HGI after adjusted for multiple cardiovascular risk factors plus HbA1c. There was significant heterogeneity among studies (I^2^ = 51%, *P* = 0.10). Random-effects models analyses showed that when adjusted for multiple cardiovascular risk factors and HbA1c, the association between HGI and risk of composite CVD became insignificant (per 1 SD increment of HGI: HR = 1.01, 95% CI = 0.93–1.10, *P* = 0. 79) (Figure 4). Similarly, there was no association between HGI and risk of all cause mortality after adjustment with HbA1c (per 1 SD increment of HGI: HR = 1.03, 95% CI = 0.96–1.10, *P* = 0.37) (Figure 5).

### Sensitivity Analyses and Subgroup Analyses

We performed several sensitivity analyses and confirmed that the results were not influenced by the use of fixed-effects models compared with random-effects models, or recalculating the HRs by omitting one study at a time.

The results of subgroup analyses for the risk of composite CVD and HGI were presented in Table 2. A high level of HGI was associated with an increased risk of composite CVD in patients without baseline CVD (HR = 1.22, 95% CI = 1.04–1.43), even after adjustment for HbA1c. However, there was no significant association observed between HGI and composite CVD in patients with a history of CVD (HR = 0.96, 95% CI = 0.88–1.06) (*P* for subgroup heterogeneity = 0.01, *I*^2^ = 84.5%). There were no significant associations for HGI and composite CVD risk among all other subgroup analyses.

**Table 2:** Subgroup analyses of the association between HGI and risk of composite CVD.

	**Number of studies**	**Per 1 SD increment of HGI**	
		**HR (95% CI)^#^**	*P* value^*^/I^**2**^
Participants age			0.61/0%
≤ 60	2	1.07 [0.81, 1.42]	
>60	2	0.99 [0.93, 1.06]	
Follow-up duration			0.61/0%
≤ 5 years	2	0.99 [0.93, 1.06]	
>5 years	2	1.07 [0.81, 1.42]	
Baseline CVD			0.01/84.5%
Yes	2	0.96 [0.88, 1.06]	
No	2	1.22 [1.04, 1.43]	

# *Adjusted for multiple cardiovascular disease and level of HbA1c*.

* *For heterogeneity among subgroups*.

*CVD, cardiovascular disease; HGI, hemoglobin glycation index; SD, standard-deviation*.

## Discussion

In this study, we included five observational studies, including more than 22,000 T2DM patients with a median follow-up duration of 5 years for meta-analysis. We found that high HGI was associated with an increased risk of composite CVD, and all cause mortality after adjustment of multiple cardiovascular risk factors. However, when further adjusted for levels of HbA1c, the association between HGI and poor prognosis was disappeared.

Although still with controversies, several mechanisms had been proposed to explain the association between HGI and the risk of CVD. First, patients with high HGI had a longer duration of diabetes and combined cardiovascular risk factors (15). Second, high HGI had been reported to be associated with subclinical target organ damage, such as coronary artery calcification (12), and carotid atherosclerosis (14). Third, high HGI is associated with activated inflammation status, which played an important role in the development of CVD (29). However, our results raised concern on these proposed mechanisms, because after adjusting for HbA1c, the association between HGI and poor prognosis was disappeared. These results indicated that the impact of HGI on CVD and all cause mortality was mainly mediating by the level of HbA1c. These finding were supported by data from the Diabetes Control and Complications Trial (DCCT), which showed that among type 1 diabetic patients, HGI was not an independent risk factor for microvascular complications, and that the effect of the HGI on risk of microvascular complications was wholly explained by the associated level of HbA1c (30).

Recently, Zhang et al. had reported a meta-analysis, which showed that for each 1-unit increment in HGI, there was a 20 and 13% increase of composite CVD and all cause mortality, respectively (31). Our analysis had several different findings compared with Zhang's report. First, we calculated the risk estimate before and after adjustingd for HbA1c, respectively, but not lumped them together, which was useful for interpreting the mediating effect of HbA1c on the association between HGI and poor prognosis. Second, we set up more strict inclusion criteria to avoid incorrect inclusion. For example, we did not include a study that reported a U-shaped association between HGI and the prognosis in patients with stroke, in which the data cannot be transformed into a continuous increment of HGI (32). We also did not include the study by Hempe et al., in which the association between HGI and risk of composite CVD or all cause mortality was not reported (33). Third, we also find that a high level of HGI was associated with an increased risk of composite CVD in patients without baseline CVD, even after adjustment for HbA1c. However, there was no significant association observed between HGI and composite CVD in patients with a history of CVD. This interesting finding raised a hypothesis that in T2DM without a history of CVD, detection of HGI may provide further information for risk stratification. However, due to the limited number of studies available, further studies are urgently needed.

Our study has several limitations. First, this is a meta-analysis based on the study level. We did not have individual participant data. Therefore, residual confounders could not be avoided. However, all the included studies had adjusted for multiple conventional cardiovascular risk factors. Second, the calculation of HGI differed among the included studies, which would cause significant heterogeneity among the studies. Third, it had been proposed that the variance in HbA1c was different in Asians and Westerns (34). However, due to the limited studies available, we did not perform subgroups analysis according to race. Further studies are needed to explore whether the associations between HGI and poor prognosis are different among different ethnicities.

## Conclusions

Our results showed that although high HGI was associated with an increased risk of composite CVD and all cause mortality after adjustment for multiple conventional cardiovascular risk factors. However, the association was mainly mediating by the level of HbA1c. Considering that HbA1c can be easily evaluated in most laboratories worldwide, and the calculation of the HGI is complicated, clinical use of HGI in T2DM cannot be routinely recommended.

## Data Availability Statement

The raw data supporting the conclusions of this article will be made available by the authors, without undue reservation.

## Author Contributions

J-dW, D-lL, YX, M-yC, and H-hC: research idea and study design. J-dW, D-lL, DS, and H-qH: data acquisition. J-dW and D-lL: data analysis and interpretation. J-dW and YX: statistical analysis. J-dW: supervision and mentorship. All authors contributed important intellectual content during manuscript drafting or revision and accept accountability for the overall work by ensuring that questions pertaining to the accuracy or integrity of any portion of the work are appropriately investigated and resolved.

## Conflict of Interest

The authors declare that the research was conducted in the absence of any commercial or financial relationships that could be construed as a potential conflict of interest.
