# Supplementary material for: Association Between Hemoglobin Glycation Index and Risk of Cardiovascular Disease and All Cause Mortality in Type 2 Diabetic Patients: A Meta-Analysis
Source: Front Cardiovasc Med. 2021 May 28;8:690689. doi: 10.3389/fcvm.2021.690689 (PMC8193090; doi:10.3389/fcvm.2021.690689)
Supplement: Supplementary file 1 [file Data_Sheet_1.DOC]

**Supplemental file 1. Quality Assessment of the Included Studies**

| **Study** | **Selection**  **(stars awarded)** | **Comparability (stars awarded)** | **Outcome**  **(stars awarded)** | **Quality (total stars)^*^** |
| --- | --- | --- | --- | --- |
| van Steen 2017 | 3 | 2 | 2 | Good (7) |
| Jin 2018 | 3 | 1 | 2 | Good (6) |
| Kim 2018 | 3 | 2 | 2 | Good (8) |
| van Steen 2018 | 2 | 1 | 2 | Good (7) |
| Ostergaard 2019 | 3 | 2 | 2 | Good (8) |


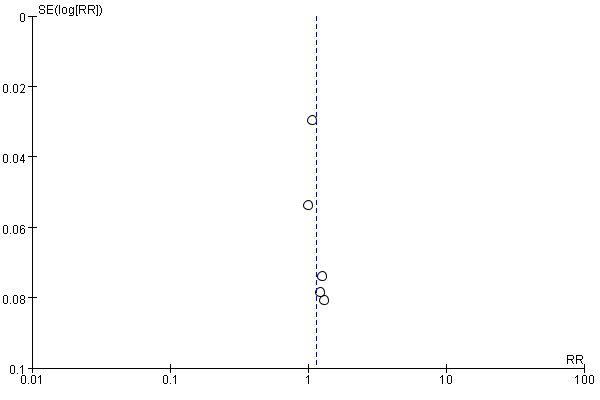


**Supplementary file 2. Funnel plot of comparison of risk of composite CVD associated with HGI.**
